# Failed suicide and deliberate self-harm: A need for specific nomenclature

**DOI:** 10.4103/0019-5545.31594

**Published:** 2006

**Authors:** P. Sarkar, F.A. Sattar, N. Gode, D.R. Basannar

**Affiliations:** * Classified Specialist and Associate Professor Department of Psychiatry, Pune University, Pune, Maharashtra; ** Senior Adviser and Professor of Psychiatry Pune University, Command Hospital (SC), Pune 411040, Maharashtra; *** Registrar Military Hospital, Jhansi 284001, Uttar Pradesh; **** Scientist ‘D’ Statistician Armed Forces Medical College, Pune 411040, Maharashtra

**Keywords:** Suicide, failed suicide, deliberate self-harm

## Abstract

**Background:**

Out of those who attempted self-harm and survived, many actually wanted to die and many did not. Presently, no distinctive nomenclature exists for these two groups, which causes difficulty in understanding as well as in management and research.

**Aim:**

To study whether there exist two such groups which are distinct and can be differentiated clinically.

**Methods:**

Seventy-eight persons who attempted self-harm were evaluated in detail by a psychiatrist. The data were recorded in an especially designed proforma which documented sociodemographic variables, psychiatric and physical illnesses, psychosocial stress factors, substance abuse, past and family history and details of suicide attempt.

**Results:**

Two groups emerged with distinct characteristics. The two groups were different in factors such as age, diagnosis, intentionality, lethality, mode, motive to kill oneself, past/family history, relation to stress, personality traits and precaution to prevent detection before and/or after the act. The group which had persons who really wanted to die but survived is suggested to be named as the ‘failed suicide’ group and the other group which had persons who did not actually want to die is suggested to be named as the ‘deliberate self-harm’ group.

**Conclusion:**

Those who cause harm to themselves but survive can be distinctly put into two groups: (i) the ‘failed suicide’ group constituting those who actually wanted to kill themselves and (ii) the ‘deliberate self-harm’ group constituting those who did not actually want to die. The criteria for distinctions are suggested.

## INTRODUCTION

Stengel^1^ identified differences between people who completed suicide and who attempted suicide but survived. He said that a degree of suicidal intent was present in both groups and those who survived were ‘failed suicide’. Kessel and Grossman^2^ found that most of those who ‘attempted suicide’ had performed their act in the belief that they were comparatively safe; that even in the heat of the moment they were aware that they would survive; and that they did not really want to commit suicide. Based on this reasoning, Kessel and Grossman proposed that ‘attempted suicide’ be replaced by ‘deliberate self-poisoning’ and ‘deliberate self-injury’.^2^ Kreitman and his co-authors^3^ introduced the term ‘parasuicide’ to refer a non-fatal act of deliberate self-injury or deliberate self-poisoning. The term parasuicide excluded the question whether death was desired or not. Morgan^4^ suggested the term deliberate self-harm (DSH) to provide a single term covering deliberate self-poisoning and deliberate self-injury. However, all these terms such as ‘attempted suicide’, ‘parasuicide’ and ‘DSH’ are still somewhat confusing, because in practice they include persons who really have the intent of killing oneself but survive the attempt.^5^

Carroll-Ghosh *et al.*^6^ make a distinction between those who ‘attempt’ and those who ‘complete’ suicide in that those who attempt are more likely to be females, under 35 years of age, use low means of lethality (e.g. wrist laceration), do it in a setting where there are high chances of rescue, and usually suffer from adjustment disorder or personality disorder. In comparison those who complete suicide are usually males, over 60 years of age, use high means of lethality (e.g. firearms, hanging), do it in a setting where there are low chances of rescue and usually suffer from mood disorder and substance abuse. Roy^7^ mentions that those who attempt and those who commit suicide represent different populations with some overlap.

In clinical practice we come across many patients who attempted serious suicidal acts and just survived out of sheer chance. Many of these patients suffer from severe mental illnesses at the time of attempting suicide. However, in the absence of a clear-cut nomenclature, patients in this group are clubbed with those who actually did not want to die. A study was thus undertaken to observe whether there exist two distinct groups of patients who are presently categorized under the common heading of DSH.

## METHODS

The study was conducted during 2001–2004 in the psychiatry department of a large general hospital in urban India, but has a major drainage from rural areas. All patients who attempted to harm themselves and came to notice were referred for psychiatric evaluation. Out of 84 patients who came to the notice during the study period, 6 declined to give consent to join the study on the ground that their so-called self-harm/poisoning was accidental and not deliberate. These 6 patients were adolescents and young adults and lethalities of harm were low.

The remaining 78 patients were interviewed by a psychiatrist who obtained a detailed history from patients, relatives, friends, employees, colleagues, eyewitnesses and various authorities–wherever applicable and at various points of time. The data were recorded in a specially designed proforma documenting the sociodemographic variables, psychiatric and physical illnesses, psychosocial stress factors, substance abuse, past and family history and details of suicide attempts. The details of suicidal attempt included number of times thought about suicide, whether impulsive or planned, intentionality, lethality, mode, whether sought help before and/or after the act and any final acts such as writing a suicide note or making a will. The intentionality and lethality were graded into three stages of severity-low, medium and high.

The patients were further assessed for any immediate or subsequent risk of attempt and appropriate treatment was offered. Diagnoses were made as per the *ICD-10 classifications for mental and behavioural disorders—diagnostic criteria for research* (DCR).^8^

## RESULTS

Out of 78 study patients, 26 were identified as those who actually wanted to kill themselves but survived accidentally. Intentionality and lethality of the attempts were quite high, most of the attempts were planned, many took precautions to prevent detection and they hardly sought any help before or after the act. Almost all of them were suffering from serious mental illnesses. Patients in this group were distributed among all ages and 66% were married.

In the other group, 85% of patients were young adults and adolescents, 75% were unmarried, 70% were suffering from stress-related illnesses with prominent unstable and histrionic traits in personality, and 20% were suffering from mild depressive episodes. The remaining 10% consisted of moderate depressive episodes, dysthymia, somatization and dissociative disorders. Table 1 gives the sample characteristics of patients relating to age, sex, marital and occupational status, education, type of family and place of residence. Table 2 shows the distribution of patients as per planning, intentionality, lethality, precaution taken to prevent detection, help-seeking before or after the act and ‘final act’.

**Table 1:** Sociodemographic distribution

		Failed suicide (*n*=26)	Deliberate self-harm (DSH) (*n*=52)
Age (years)	15-20	2	14
	21-30	12	29
	31-40	8	9
	41-50	4	0
Sex	Male	18	27
	Female	8	25
Marital status	Single	9	41
	Married	17	11
Employment status	Employed	18	13
	Unemployed	2	4
	Student	-	33
	Housewife	6	2
Education	Illiterate	Nil	Nil
	V standard	3	2
	X standard	10	11
	College	13	39
Type of family	Nuclear	17	44
	Joint	9	8
Place of residence	Urban	9	36
	Rural	17	16

Age: There is a significant association between status and age (p<0.01) Sex: There is no significant association between disease status and sex (p>0.05) Marital status: There is a significant association between disease status and marital status (p>0.01) Educational status: There is no significant association between disease status and educational status (p>0.05) Type of family: There is no significant association between disease status and type of family (p>0.05) Urban/rural status: There is a significant association between disease status and urban/rural status (p<0.01)

**Table 2:** Distribution as per planning, intentionality, lethality, precautions to prevent detection, help-seeking and final acts

		Failed suicide (*n*=26)	Deliberate self-harm (DSH) (*n*=52)
Planning	Not planned	6	40
	Planned	20	12
Intentionality	High	26	1
	Medium	0	13
	Low	0	38
Lethality	High	19	1
	Medium	6	6
	Low	1	45
Took precaution to prevent detection	Yes	20	9
	No	6	43
Sought help before the act	Yes	0	9
	No	26	43
Sought help after the act	Yes	2	46
	No	24	6
Final acts (suicide note, will)	Yes	2	1
	No	24	51

Planning: There is a significant association between disease status and planning (p<0.01) Intentionality: There is a significant association between disease status and intentionality (p<0.01) (low and medium clubbed together for analysis) Lethality: There is a significant association between disease status and lethality (p<0.01) (low and medium clubbed together for analysis) Took precaution: There is a significant association between disease status and precaution taken (p<0.01) Sought help before: There is a significant association between disease status and seek help before (p<0.01) Sought help after: There is a significant association between disease status and seek help after (p<0.01) Final acts: There is no significant association between disease status and final acts (p<0.01)

Table 3 shows the psychiatric diagnosis as per the ICD-10 DCR. Table 4 shows the mode of attempt of the failed suicide group and Table 5 shows the mode of attempt of the DSH group. Table 6 depicts distinctions between failed suicide and DSH.

**Table 3:** Psychiatric diagnoses as per ICD-10 DCR

Diagnosis	Failed suicide	Deliberate self-harm (DSH)
Severe depressive episode	17	0
Moderate depressive episode	3	4
Mild depressive episode	-	10
Schizophrenia	1	0
Post-schizophrenic depression	3	0
Dysthymia	-	2
Somatization disorder	-	1
Dissociative disorder	-	1
Alcohol-dependence syndrome with adjustment disorder	1	1
Adjustment disorders with emotionally unstable/histrionic personality traits/disorder	1	33
**Total**	**26**	**52**

**Table 4:** Modes of attempts of the ‘failed suicide’ group

S. No.	Mode of attempt	Male	Female	Diagnosis
1.	Gunshot neck injury	1	-	Severe depressive episode
2.	Gunshot precordium injury	1	-	Severe depressive episode
3.	Hanging–became unconscious	3	2	Severe depressive episode-3
				Moderate depressive episode
				Post-schizophrenic depression
4.	Hanging–did not become unconscious	-	1	Severe depressive episode
5.	Stab injury abdomen, then jumped into a river	1	-	Severe depressive episode
6.	Cut major artery of arm	-	1	Schizophrenia
7.	Stab injury abdomen	2	-	Severe depressive episode-2
				Post-schizophrenic depression
8.	Slit throat and multiple deep cuts all over the body	1	-	Severe depressive episode
9.	Slit throat	2	-	Severe depressive episode
10.	Organophosphorous poisoning	2	-	Alcohol-dependence syndrome with adjustment disorder
				Alcohol-dependence syndrome with emotionally unstable personality disorder
11.	Jumped from third floor	-	3	Severe depressive episode-2
				Post-schizophrenic depression-1
12.	Self-immolation	-	2	Severe depressive episode-2
13.	Consumed acid	-	1	Moderate depressive episode
14.	Consumed diazepam (5 mg) 200 tablets	1	-	Moderate depressive episode
15.	Touching live electric wire of 220 volt	1	-	Severe depressive episode
16.	Jumping into a deep lake (non-swimmer)	-	1	Severe depressive episode

**Table 5:** Modes of attempt of the ‘deliberate self-harm’ (DSH) group

S.No.	Mode of attempt	*n*	Male	Female
1.	Wrist cut-superficial	11	5	6
2.	Cut the skin at few places–superficial	4	2	2
3.	Benzodiazepine overdose	12	5	7
4.	Consumed combination of available drugs	6	2	4
5.	Consumed rat poison	7	3	4
6.	Consumed cockroach poison	2	-	2
7.	Consumed weak acid	4	2	2
8.	Consumed dimethyl pthalate (mosquito repellent)	4	4	-
9.	Consumed kerosene oil	1	1	-
10.	Hanging-became unconscious (impulsively hanged himself over a trivial issue–emotionally unstable personality disorder)	1	1	-
	**Total**	**52**	**25**	**27**

**Table 6:** Distinctions ‘failed suicide’ and ‘deliberate self-harm’ (DSH)

		Failed suicide	DSH
1.	Age	Distributed in all ages	Mostly in adolescentsand young adults
2.	Illness	Severe depression and major psychiatric illnesses	Stress-related illnesses
3.	Intentionality	High-medium	Low
4.	Lethality	High-medium	Low
5.	Motive emotions/help-seeking	To die	To drain pent-up
6.	Stress	May not be significant	Yes
7.	Mode	Significantly harmful	Not much harmful
8.	Past history of similar attempt	May be present	May be present
9.	Family history of suicide/serious attempt	May be present	Unlikely
10.	Presence of prominent histrionic, unstable traits in personality	May be present	Most probable
11.	Planning	Usually planned	Usually impulsive
12.	Precaution to prevent detection before and/or after the act	Usually taken	Usually not taken

## DISCUSSION

Of the two distinct groups that emerged, one group consisted of 26 patients who were suffering from serious psychiatric illnesses—17 from severe depressive episodes, 3 from moderate depressive episodes, 3 from post-schizophrenic depression, 1 from schizophrenia and 1 from alcohol-dependence syndrome with adjustment disorder and 1 from adjustment disorder with emotionally unstable personality. This group was evenly distributed in all ages. Eighty per cent of the attempts were planned and 100% had high intentionality. Lethality of the attempts was high in 70% of cases and medium in 20% of cases. Eighty-two per cent of patients took precautions to prevent detection, none of them sought help before the act, and only 8% sought help after the act. Eighty-five per cent of patients in this group would have died and would have been counted under the ‘those who complete’ group of Carroll–Ghosh *et al*.^6^ but had accidentally survived and under the existing classification system are being grouped under ‘those who attempt’ group. They are actually the ‘failed suicide’ group of Stengel.^1^

Fifty-two patients, who formed the second group, were different from the other 26 patients. In the second group, the patients were mostly adolescents and young adults, 80% were unmarried, mostly suffered from stress-related illnesses, with underlying unstable and/or histrionic personality traits/disorders, few had mild depressive disorders and none were suffering from severe depressive episodes. Intentionality was low in 80% of cases and moderate in 15% of cases. Lethality was low in about 88% of cases and medium in 10% of cases and high only in 1 patient. None but one of the attempts had high intentionality. Eighty-seven per cent of the patients did not take any precautions to prevent detection and 90% sought help after the attempt. The modes were also non-violent; 27% of them cut wrists or cut other sites—all generally superficial cuts; the remaining consumed benzodiazapines, other available medicines, rat and cockroach poison, kerosene oil, mosquito repellents and weak acids such as phenol. None of them consumed the above items in excess amounts and common sense suggests that the amounts were inadequate to kill a human being. A majority of them soon disclosed the attempts to others. This group of patients, except 1 patient where intentionality might be high, did not have any convincing history or clinical finding that indicates that he/she really wanted to die. Analyses showed that a majority of these were impulsive attempts, under some stress, against the background of unstable personality; some attempts were attention-seeking and others sought to ventilate pent-up emotions or to manipulate an environment with an obvious conscious gain.

Of the two interesting findings in the study, the first is that only 2 patients from the ‘failed suicide’ group and 1 patient from the DSH group wrote a suicide note and none had written a will. Maybe writing a suicide note or a will is not prevalent in this part of the world. The second interesting finding is that while 34% of patients of the failed suicide group had an urban background, 68% of the DSH group had an urban background. Considering that 70% of the Indian population resides in villages, this finding assumes importance. Is the DSH mostly an urban phenomenon? As most of the group is constituted of young people, mostly students, is DSH limited to this population? Do chances of DSH reduce with increasing age, when their personality becomes more stable and they get better equipped to handle stress and be less dramatic?

Thus, there emerged two groups of patients with distinct characteristics. The first group is different from the second group in diagnoses, intentionality, lethality, mode, age and, most importantly, the motive. The first group of patients really wanted to kill themselves, whereas the second group did not. This differentiation is important as their management as well as course and prognosis of survivors are likely to be different. Unless different nomenclatures are used for these two groups, it becomes difficult for a scientific study to elicit relevant information. For example, a valuable long-term follow-up study of 11,583 patients who presented to a general hospital in Oxford between 1978 and 1997 for repetition of DSH and subsequent suicide risk finds that 39% repeated DSH.^9^ Other western studies also find that repetition of DSH is associated with an increased risk of eventual suicide.^10^–^12^ However, in the absence of a clear distinction between the ‘failed suicide’ and the DSH groups of patients, these studies are unable to throw light on who are the repeaters and which repeater ultimately completes suicide. A person suffering from emotionally unstable and/or histrionic personality disorder may repeat a DSH several times without dying, whereas a person suffering from recurrent severe depression may complete it in the next attempt.

In a study to differentiate the psycho-sociodemographic profile of suicide attempters and suicide completers, conducted in a general hospital setting in Kerala, 750 cases of suicide attempters were compared with 689 suicide victims.^13^ Many of the differences in the psycho-sociodemographic profile of suicide attempters and completers reported from western countries could not be replicated in this study from India. Similar observations have been reported from India in suicide^14^,^15^ and attempted suicide.^16^–^19^ These studies repeatedly emphasized that those who attempt suicide carry the risk of repeating it and that studies from India on suicide attempters and completers show different psycho-sociodemographic profile from those of the West. Thus, a psychiatrist is not alerted about a specific group of patients who may be more vulnerable to complete the act.

The present study re-emphasizes that there is an immediate need to employ two different nomenclatures for these two distinct groups of patients. One group is constituted of patients that actually wanted to die, i.e. the group of 26 patients in the present study. This group should be separated from the second group of 52 patients who actually did not want to die.

The term ‘failed suicide’ suggested by Stengel^1^ (which is self-explanatory) for the former group and ‘deliberate self-harm’ (DSH) suggested by Morgan^4^ for the latter group are being proposed as the nomenclatures. This distinction will help clinicians to identify the risk in their patients and consequently in their management and follow-up. Research in this field will also become more specific.

A limitation of the above proposal may be, as some may argue, that there might be overlaps in these two groups. Further research, after adopting such nomenclature, will throw light on this aspect. Another limitation of the study is that the sample size is not large enough. However, it is a 4-year long prospective study, conducted in a general hospital setting where all types of patients come and hence the sample should be adequate enough to arrive at a conclusion. Moreover, each case was analysed in detail.

It may be asked how does one separate the two groups in actual practice? Is such separation possible? What are the suggestions to achieve such separation? Are there people who fall in between where dichotomous decision-making is difficult? The answer to the above questions, as the present study brings out, is that it is possible to separate these two groups based on the criteria shown in Table 6. The two groups are clinically quite distinct and chances of overlap are minimal.

## CONCLUSION

Many of those who attempt self-harm and survive, actually wanted to die, and many did not. Presently, no such distinctive nomenclature exists for these two groups. This study was conducted to find out whether such distinction is possible. There emerged two distinct groups: (i) those who actually wanted to die but survived (failed suicide) and (ii) those who did not want to die (deliberate self-harm). Such distinction is likely to help in understanding the clinical situation and management of patients as well as in future research.

## References

[1] Stengel E (1952). Enquiries into attempted suicide. Proc R Soc Med.

[2] Kessel N, Grossman G (1965). Suicide in alcoholics. Br Med J.

[3] Krietman N (1977). Parasuicide.

[4] Morgan HG (1979). Death wishes? The understanding and management of deliberate self harm.

[5] Kerkhof A JFM, Arensman E, Gelder MG, Lopez Ibor JJ, Andresen NC (2000). Attempted suicide and deliberate self harm: Epidemiology and risk factors. New Oxford textbook of psychiatry.

[6] Carroll-Ghosh T, Victor BS, Bourgeors JA, Hales RE, Yudof Sky SG (2003). Suicides. Textbook of clinical psychiatry.

[7] Roy A, Sadock BJ, Sadock VA (2000). Suicide. Comprehensive textbook of psychiatry.

[8] World Health Organization (1992). The ICD-10 classification of mental and behavioural disorders. Diagnostic criteria for research.

[9] Zahl DL, Hawton K (2004). Repetition of deliberate self-harm and subsequent suicide risk: Long term follow-up study of 11583 patients. Br J Psychiatry.

[10] Hawton K, Fagg J (1998). Suicide and other causes of death, following attempted suicide. Br J Psychiatry.

[11] Tezedor HC, Digz A, Castillon JJ (1999). Attempted Suicide: repetition and survival-findings of a follow-up study. Acta Psychiatr Scand.

[12] Owens D, Horrocks J, House A (2002). Fatal and non-fatal repetition of self harm Systematic review. Br J Psychiatry.

[13] Kumar PNS (2004). An analysis of suicide attempters and suicide completers in Kerala. Indian J Psychiatry.

[14] Sathyavathi K, Murthi Rao DLN (1961). A study of suicide in Bangalore. Transactions of All India Institute of Mental Health.

[15] Shukla GD, Verma BL, Mishra DN (1990). Suicide in Jhansi city. Indian J Psychiatry.

[16] Kumar PNS (1998). Age and gender related analysis of psychosocial features in attempted suicide. Indian J Psychiatry.

[17] Narang RL, Mishra AP, Mohan N (2000). Attempted suicide in Ludhiana. Indian J Psychiatry.

[18] Kristy NP, Rawal NK, Jawedkar AN (2002). A study of attempters of deliberate self harm. Ind Psychiatry J, India.

[19] Kumar PNS (2000). A descriptive analysis of methods adopted, suicide intent and causes of attempting suicide. Indian J Psychol Med.

